# Decoding the Pluripotency Network: The Emergence of New Transcription Factors

**DOI:** 10.3390/biomedicines1010049

**Published:** 2013-12-16

**Authors:** Kai Chuen Lee, Wing Ki Wong, Bo Feng

**Affiliations:** 1 Key Laboratory for Regenerative Medicine, Ministry of Education, School of Biomedical Sciences, Faculty of Medicine, the Chinese University of Hong Kong, Room 105A, 1/F, Lo Kwee-Seong Integrated Biomedical Sciences Building, Area 39, Shatin, N.T., Hong Kong, China; E-Mails: bolkcad@gmail.com (K.C.L.); winisama@gmail.com (W.K.W.); 2 SBS Core Laboratory, Shenzhen Research Institute, the Chinese University of Hong Kong, 4/F CUHK Shenzhen Research Institute Building, No.10, 2nd Yuexing Road, Nanshan District, Shenzhen 518057, China

**Keywords:** pluripotency, embryonic stem cell, transcriptional network, transcription factor

## Abstract

Since the successful isolation of mouse and human embryonic stem cells (ESCs) in the past decades, massive investigations have been conducted to dissect the pluripotency network that governs the ability of these cells to differentiate into all cell types. Beside the core Oct4-Sox2-Nanog circuitry, accumulating regulators, including transcription factors, epigenetic modifiers, microRNA and signaling molecules have also been found to play important roles in preserving pluripotency. Among the various regulations that orchestrate the cellular pluripotency program, transcriptional regulation is situated in the central position and appears to be dominant over other regulatory controls. In this review, we would like to summarize the recent advancements in the accumulating findings of new transcription factors that play a critical role in controlling both pluripotency network and ESC identity.

## 1. Introduction

The culturing of pluripotent embryonic stem cells (ESC) began in the 1980s, when they were established by explanting the inner cell mass (ICM) from mouse embryos at blastocyst stage [1]. The ICM serves as the origin of all somatic tissues and ultimately develop into an embryo. ESCs have inherited the same property of pluripotency from ICM [1]; thus, they can differentiate into all three germ layers (ectoderm, endoderm and mesoderm) while proliferating robustly in culture. This feature has rendered ESCs great potential in scientific research and medical treatment. During 2006–2007, Takahashi *et al.* reprogrammed human and mouse somatic cells into ESC-like pluripotent cells (termed induced pluripotent stem cells or iPSC), simply by direct transduction of ESC transcription factors Oct4, Sox2, Klf4, and c-Myc [2,3]. These groundbreaking studies have opened possibilities to generate patient-specific pluripotent cells for therapy. Its clinical relevance aroused great interest in stem cell research and attracted much attention to the investigation of molecular regulations underlying the fascinating property of pluripotency.

From the original setting of mouse ESC culture, scientists identified the active components leukaemia inhibitory factor (LIF) and bone morphogenic proteins (BMP), which maintain the pluripotency in ESCs through activating the Jak/Stat3 signaling pathway and inducing the *inhibitors of differentiation (Id)* genes, respectively [4,5,6]. In a later study, inhibition of protein kinases ERK1/2 and GSK3β using small-molecule inhibitors (termed “2i”) was found to safeguard mouse ESC in a ground state of pluripotency, while abolishing the requirement of LIF and BMP [7]. Derivation of human ESCs was another milestone discovery in stem cell biology. When human ESCs were first established in 1998 [8], they were found to exhibit profound differences in morphology and proliferation rates from mouse ESCs. Moreover, human ESCs depend on fibroblast growth factor (FGF) and transforming growth factor beta (TGFβ)/Activin/Nodal signal pathways, but not LIF and BMP signaling, to maintain their self-renewal and pluripotency. Collectively, these signal requirements by mouse and human ESCs provided the basic foundation for subsequent understanding of the molecular regulations in pluripotency maintenance.

To date, massive investigations have been conducted to dissect the pluripotency network. The unique regulatory circuitry in pluripotent stem cells has been gradually unraveled with accumulating discoveries about the core and ancillary transcription factors [9,10], epigenetic modulation complexes that facilitate the maintenance of open chromatin structure [11,12], microRNAs (miRNA) that direct the mRNA degradation or disrupt the translation of pluripotency factors [13,14], as well as the large intergenic non-coding RNAs (lincRNAs) that affect a broad range of gene expressions in ESCs [15]. Among the various regulations that orchestrate the cellular pluripotency program, transcriptional regulation holds a central position and plays a dominant role. In this review, we would like to summarize the recent advancements in the understanding of transcriptional regulations related to pluripotency maintenance, with special focus on the accumulated findings concerning newly identified transcriptional factors which play critical roles in maintaining pluripotency besides the *Oct4*, *Sox2* and *Nanog* core complex.

## 2. Core Pluripotency Network

### 2.1. Oct4, Sox2 and Nanog

Oct4 is a POU family transcription factor, which acts as a key regulator to govern pluripotency and ESC identity [16]. Its expression is exclusively restricted to early embryos at cleavage stages, ICM in blastocysts and germ cell lineage in later development [16]. *Oct4*-deficient embryos can develop to the blastocyst stage, but the ICM cells are not pluripotent and cannot form ESCs in culture [16]. In ESCs, the expression of *Oct4* is under stringent control. Reduced expression causes ESC to differentiate into trophoectoderm, whereas enhanced expression induces differentiation to primitive endoderm lineage [17]. Sox2 is a high mobility group (HMG) domain containing transcription factor, which is also expressed in mouse embryos at cleavage stages and ICM of blastocysts [18]. Similarly to *Oct4*, *Sox2*^−/−^ mouse embryo showed a primary defect in ICM and cannot give rise to ESCs [18]. Direct knockdown of *Sox2* in ESC causes rapid differentiation. Remarkably, Sox2 interacts directly with Oct4 to form a complex that recognizes Oct4-Sox2 regulatory elements in downstream target gene promoters to regulate their transcription [18]. Interestingly, family members of Oct4 and Sox2 could also interact directly and form various Oct-Sox complexes [19,20]. The tightly regulated expression of *Oct* and *Sox* genes as well as selective binding to different target sites by various Oct-Sox partnerships have been found to play critical roles in controlling the self-renewal and differentiations of ESCs [20,21]. 

Nanog is identified as another important pluripotency factor due to its unique expression pattern in undifferentiated cells [22,23]. Genetic deletion of *Nanog* caused early embryonic lethality, and ICM from *Nanog*^−/−^ mouse embryos failed to give rise to ESCs [24]. Moreover, in mouse ESCs, elevated *Nanog* expression was found to alleviate the requirement of LIF for maintaining their self-renewal [22,23]. However, unlike Oct4 and Sox2, Nanog did not exhibit strong reprogramming capacity [2,3]; and *Nanog*^−/−^ ESCs can partially self-renew and remain largely pluripotent [25]. Hence, Nanog was defined as a regulator that acts mainly in construction of pluripotent states rather than the housekeeping of pluripotency. 

### 2.2. Core Regulatory Circuitry for Pluripotency

Oct4, Sox2 and Nanog converge and form the primary, yet central, network that governs the robust pluripotent state. They regulate themselves by binding to their own promoter as well as the promoters of the other two, forming a feed-forward auto-regulatory circuitry that stabilizes the core regulatory circuitry in the entire pluripotency network [26,27]. In genome wide binding analyses through chromatin-immunoprecipitation (ChIP) coupled with gene chip array (ChIP-on-chip) or paired-end ditag sequencing (ChIP-PET), these three factors were found to co-occupy many of their target genes and exhibit a substantial overlapping of their binding maps in ESC genomes [9,10]. Further comparison with microarray profiles showed that most of these co-target genes were up-regulated in pluripotent state and down-regulated upon differentiation [9,10], conferring the dominant role of these three factors in activating ESC-specific genes. In addition, Oct4, Sox2 and Nanog were also found to occupy the promoter of silenced genes, which are mainly differentiation promoting genes involved in lineage specification [9,10]. This suggests that the core factors also form co-regulatory feedback loops to repress or suspend the expression of differentiation genes.

Extended genome binding analyses on other ESC factors using ChIP coupled with high-throughput sequencing (ChIP-seq) further revealed that Oct4, Sox2 and Nanog often cooperate with other transcription factors or co-activators to modulate gene activation/repression in ESCs [28,29]. The number of transcriptional regulators binding to a promoter also dictates gene expression in ESCs. Promoters that are co-occupied by more than four transcription factors tend to be active in the pluripotent cells and silenced in differentiated cells, whereas promoters bound by a single factor tend to be inactive in ESC and induced upon differentiation [28,29]. Furthermore, genes activated by these core factors were often found to activate the expression their activators, thus forming feed-forward regulations to maintain the pluripotency [28,29,30].

Besides the well-studied Oct4, Sox2 and Nanog, extensive studies have been carried out to identify new transcription factors in ESCs and examine the interrelationship between these factors and their connection to the entire pluripotency network. Below we will discuss these adjunct factors and their role in pluripotency maintenance. 

## 3. Nuclear Receptors and Pluripotency

Nuclear receptors (NRs) are a large family of ligand-regulated transcriptional factors, which are involved in various functions, including homeostasis, reproduction, development and metabolism [31]. There are 48 NRs identified in human [32] and 49 identified in mouse [33]. All the NRs share conserved common structures. Their *N*-terminal region is the highly variable A/B domain that contains at least one transcriptional activation function 1 domain (AF-1) and several autonomous activation domains (AD). In addition, there is a conserved zinc finger DNA binding domain (DBD), a hinge region and the *C*-terminal ligand-binding domain (LBD) that overlaps with the second activation function 2 domain (AF-2). The AF1 is constitutively active in most cell types while the AF2 activity is ligand dependent [34,35]. The nuclear receptors without known ligands are usually referred as orphan nuclear receptors.

Recent studies have identified orphan nuclear receptors Esrrb, Nr5a2, Dax1 and GCNF for their involvement in pluripotency maintenance [36,37]. Since nuclear receptors in ESCs have been intensively reviewed recently [38], here we will only focus and recapitulate their function in regulating (or being regulated by) the core pluripotency factors at a molecular level.

### 3.1. Esrrb

Estrogen-related receptor (Esrr) α, β and γ showed themselves to be close homologues to the estrogen receptor (ER) but do not respond to estrogen. Instead, they are constitutively active in regulating transcription without response to known ligands. Among them, Esrrb (Estrogen-related receptor β) is closely related to pluripotency maintenance. *Esrrb*^−/−^ mouse embryos died at E10.5 due to placental defect [39]; when the placental defects were complemented with those of wild-type tetraploid embryos, proliferation of primordial germ cells (PGC) was found to be significantly reduced in the *Esrrb*^−/−^ mutants [40]. Although these studies showed that *Esrrb* was not directly involved in the formation of pluripotent ICM cells *in vivo*, gene silencing via RNAi approaches robustly demonstrated that *Esrrb* is essential to the maintenance of mouse ESCs in culture [10,41,42]. Furthermore, Esrrb was found to promote reprogramming of somatic cells into pluripotent state, by replacing Klf4 and functioning collectively with Oct4 and Sox2 to generate iPSCs [43]. These data established Esrrb as a crucial player for pluripotency maintenance.

Detailed investigation found that Esrrb directly interacts with Oct4, Sox2 and Nanog, providing insightful evidence on how Esrrb fits into the pluripotency transcription network in ESCs. Esrrb interacts with Oct4 protein [44], and activates *Nanog* expression by localizing to the Oct4-Sox2 elements as well as degenerate estrogen-related receptor DNA-binding elements in *Nanog* proximal promoter [44]. Esrrb also interacts directly with Nanog protein [41]. There are two Esrrb binding sites and one Nanog binding site in mouse *Oct4* promoter. Both Nanog and Esrrb were found to be required to activate *Oct4* expression [41]. In addition, Esrrb also cooperates with another core pluripotency factor, Sox2, to regulate gene expression. Employment of a motif-discovery tool called Fexcom identified the presence of Esrrb-Sox2-DNA ternary complex. Similar to the Oct4-Sox2 element, the Esrrb-Sox2 element possesses a spacer of 2 to 8 bp [45]. A recent study demonstrated that the full function of Esrrb requires the presence of its coactivator, Ncoa3 (Nuclear receptor coactivator 3). Ncoa3 interacts with Esrrb through its AF-2 portion of LBD to trigger downstream gene transcription and mediate its role in maintaining ESC [46]. Ncoa3 recruitment to target genes is Esrrb-dependent, as Ncoa3 alone could not support the self-renewal capacity of ESCs [46]. Concomitantly with Esrrb, Ncoa3 is also required for the induction of pluripotency upon reprogramming [46]. 

In return, *Esrrb* has been identified to be a direct target of Nanog [47]. Direct binding of Nanog to *Esrrb* promoter recruits RNA polymerase II to strengthen its expression [47]. It was found that Esrrb can replace Nanog in sustaining ESC self-renewal in the absence of LIF [47,48]; however, the maximal effect of Esrrb could only be achieved in the presence of Nanog. Furthermore, investigations found that *Esrrb* is also a downstream target of Tcf3 upon GSK3 inhibition. Both Tcf3 and β-catenin are required to induce *Esrrb* expression for maintaining ESC self-renewal [48], suggesting *Esrrb* is a downstream effector of WNT signaling in ESCs.

### 3.2. Nr5a2

Nr5a2 (nuclear receptor subfamily 5, group A, member 2/liver receptor homologue-1, Lrh-1) exhibits broad expression during the morula and epiblast stages of development. Its genetic ablation causes mouse embryos to die at the epiblast stage around E6.5–E7 [49]. ICM formation in the mutant embryos appeared unaffected, but exhibited a premature loss of *Oct4* expression [50]. Consistently, knockout or silencing of *Nr5a2* in ESCs did not induce differentiation immediately, but resulted in a reduced expression of *Oct4* and *Nanog* [50,51]. In 2010, Heng *et al.* discovered that Nr5a2 promoted somatic cell reprogramming and could replace Oct4 to generate iPSCs [51]. A later study by Guo *et al.* demonstrated that introduction of Nr5a2 could convert mouse epiblast stem cells (EpiSCs), which were derived from postimplantation embryos at E5.5, to the naïve pluripotency state similar to mouse ESCs [52]. These findings showed that Nr5a2 plays an important role in framing the pluripotent state. 

Molecular analysis has shown that Nr5a2 regulates *Oct4* expression by binding to its proximal promoter and upstream proximal enhancer [50]. A study by Kelly *et al.* substantiated that Nr5a2 regulates *Oct4* through interacting with another orphan nuclear receptor, Dax1 [53]. Moreover, a recent study provided evidence that Nr5a2 could activate *Oct4* by synergistically binding to the *Oct4* promoter with retinoic acid receptor gamma (RARγ) [54], which in turn promotes rapid and efficient reprogramming in combination with the Yamanaka’s four factors. Heng *et al.* has also detected direct interaction between Nr5a2 and Nanog through co-immunoprecipitation (Co-IP) [51]. Collectively, these data suggest that Nr5a2 regulates *Oct4* expression through interacting with other factors, such as Dax1, RARγ and Nanog; whereas, the relationship among these Nr5a2 binding partners still remains unclear. Besides activating *Oct4*, Nr5a2 was also found to regulate *Nanog* expression to promote ESC maintenance and somatic cell reprogramming [51]. ChIP analysis showed that Nr5a2 binds to the *Nanog* enhancer and regulates its expression directly [51]. Wu *et al*. further reported that Nr5a2 activates *Nanog* expression by binding to the *Nanog* promoter and, meanwhile, recruiting histone acetyltransferase CREB binding protein (CBP) and histone arginine methyltransferase CARM1 to establish active epigenetic marks on it [55]. 

In support of the positive feed-forward regulatory model proposed by large scale mapping analyses in ESCs [28,29], *Nr5a2* is found to be a direct target of Oct4, especially the *O*-GlcNAcylated active form of Oct4 protein [56]. In addition, similar to *Esrrb*, *Nr5a2* has also been identified as a direct target gene of β-Catenin and Tcf3 down-stream of the WNT signaling pathway [57]. Studies of the three-dimensional structures of Nr5a2 and β-Catenin showed their direct protein-protein interaction and proposed a potential co-activation mechanism [58]. 

### 3.3. Dax1

Dax1 (Nr0b1/Ahch) is characterized as an atypical orphan nuclear receptor that lacks a prospective DNA binding domain [59]. Instead, it possesses three LXXLL domains that mediate protein-protein interaction in the *N*-terminal [60]. During early developmental stages, *Dax1* is expressed in the morula and the blastocyst where cells are pluripotent [61], suggesting a role of Dax1 in regulating pluripotency. Indeed, *Dax1* is enriched in undifferentiated ESCs and is repressed upon differentiation. Silencing of *Dax1* via RNAi in wild type ESC causes immediate differentiation and reduced viability [37]. Interestingly, increased *Dax1* level has also been reported to cause ESC differentiation [62]. This suggests that Dax1 acts as both a transcriptional coactivator and corepressor [63,64], whose expression level determines itself to be a transcriptional repressor or activator [63].

Detailed analysis showed that Dax1 cooperated with Nr5a2 or Oct4 proteins to control *Oct4* transcription. Precise interaction motif between Nr5a2 and Dax1 was resolved by the crystallography analysis of their complex, showing the interaction through PCFXXLP, a repressor motif conserved among all members of the Nr0b1 subfamily [64]. In addition, Dax1 was found to associate with Oct4 proteins through the POU-domain [62]. In both situations, Dax1 is believed to regulate *Oct4* expression through modulating the activity of Nr5a2 or Oct4 proteins, which bind directly to the corresponding DNA motifs in the *Oct4* promoter and enhancers. Besides Nr5a2 and Oct4, Dax1 was also identified to interact with Nanog in a proteomics analysis [65], yet its functional significance has not been addressed. 

On the other hand, *Dax1* is tightly regulated by the core pluripotency factors in combination with signaling pathways. Stat3 and Oct4 regulated *Dax1* transcription through a putative binding site at −158 bp and +2054 bp, which are located in *Dax1* promoter and an intronic region respectively [66]. Whereas, Esrrb and Sox2 maintained *Dax1* expression though binding to the Esrrb-Sox2 motif in *Dax1* promoter [45]. Depletion of Sox2 in ESCs induced down-regulation of *Dax1* [67]. In addition, Nr5a2 and Nanog were found to regulate *Dax1* expression through binding to its promoter at −128 site or to its first intron around +2770 site, respectively [68]. Overexpression and depletion of either Nr5a2 or Nanog in mouse ESCs result in an alteration of *Dax1* expression [68]. Furthermore, *Dax1* expression was apparently affected by pharmacological activation of β-catenin in mouse ESCs [24,37], suggesting that, besides *Esrrb* and *Nr5a2*, *Dax1* could be another downstream target of the WNT signaling in ESCs. 

### 3.4. GCNF

*GCNF* (germ cell nuclear receptor/Nr6a1) exhibits broad expression throughout embryonic development and adulthood. Genetic ablation of *GCNF* caused embryonic lethality in mice, in which *Oct4* expression was no longer restricted to the germ cell lineage. *GCNF*^−/−^ ESCs showed no defect in self-renewal [69], but exhibited deficiency in differentiation due to loss of repression on pluripotency genes, including *Oct4* and *Nanog* [69,70,71,72]. This observation confers a role of GCNF in repressing *Oct4* and *Nanog* expressions [73]. Interestingly, GCNF represses *Oct4* expression through binding to an evolutionarily conserved DR0 element (direct repeats with a zero base pair spacing) located in its proximal promoter, which was also recognized by Nr5a2 for activating *Oct4* transcription [50]. The binding of GCNF and Nr5a2 to the same site suggests a reciprocal regulatory model for *Oct4* expression by these two proteins, *i.e.*, GCNF replaces Nr5a2 to bind to *Oct4* promoter and repress its expression upon ESC differentiation [50]. In a later study, GCNF was also found to repress *Nanog* expression through binding to the same DR0 element located in its proximal promoter [69,73], whereas Nr5a2 was found to activate *Nanog* expression by binding to its enhancer [51]. It is worth investigating if Nr5a2 and GCNF regulate *Nanog* expression through a similar reciprocal regulatory mechanism. 

Based on its important function in repressing pluripotency genes, demethylation of *GCNF* gene has been suggested as a marker for successful reprogramming [72]. Improper reprogramming of *GCNF* locus can lead to defects in subsequent differentiation. The *GCNF*^off^ iPSCs (showing a loss of *GCNF* expression at 1.5 days after differentiation) behaved similarly to pluripotent cells except for the reduced capacity to differentiate into all lineages. *GCNF*^off^ iPSCs were unable to give rise to chimeras after blastocyst injection. Re-introduction of GCNF could rescue the repression of *Oct4* in *GNCF*^off^ iPSCs upon differentiation [72].

## 4. Kruppel-Like (Klf) Transcription Factors

The Klf family is a set of zinc finger transcription factors, which consist of 17 family members involved in various biological processes [74]. Common to all the family members is the presence of *C*-terminal tandem zinc finger motifs known for DNA binding [75,76]. Among all the family members, Klf2, Klf4 and Klf5 are expressed at a high level in ESCs but decrease upon differentiation [77]. Strong evidence demonstrated that these three Klfs play overlapping roles in maintaining ESC self-renewal [77], and they were exchangeable in reprogramming somatic cells into iPSCs [43,78].

Klf4 is the first Klf factor highlighted in stem cell biology since its discovery in facilitating somatic cell reprogramming [2]. Since then, its function in ESCs has been intensively investigated. First, Klf4 was found to play a pivotal role in maintaining *Nanog* expression. Direct binding of Klf4 to *Nanog* promoter has been reported in both human and mouse ESCs [77,79]. In mouse ESCs, a common binding motif for Klf2, Klf4 and Klf5 has been identified at the distal enhancer of *Nanog* [77]. Either mutation in this motif or triple knockdown of *Klf2*, *Klf4* and *Klf5* severely impeded the transcriptional activity of the *Nanog* enhancer. Moreover, Klf4 was found to interact directly with Oct4 and Sox2 proteins through its conserved zinc finger motif to activate *Nanog* expression in mouse ESCs [80]. A defective Oct4-Sox2-Klf4 complex interfered with normal self-renewal of ESCs and inhibited reprogramming [80]. Collectively, these findings indicate that *Nanog* is a direct target activated by Klf4, which explains Klf4’s ability to retain the pluripotent state and prevent differentiation of embryoid bodies upon its ectopic expression [81,82]. Similarly, Klf4 could regulate human *Nanog* expression by direct binding to a conserved motif on *Nanog* proximal promoter [79]. Cooperation of Klf4 with Pbx1, Oct4 and Sox2 was found to exhibit synergistic activity to maintain *Nanog* transcription in human ESCs [79]. 

Another important target of Klf4 is *Esrrb*. ChIP assay has identified common binding regions for Klf2, Klf4 and Klf5 in the *Esrrb* upstream regulatory region [77]. Triple knockdown of *Klf2*, *Klf4* and *Klf5* severely impeded the expression of *Esrrb* [77], suggesting that *Esrrb* is a direct downstream target of these three Klfs. In support of this notion, Esrrb was found to replace Klf4 to reprogram mouse fibroblasts into iPSCs in the presence of Oct4 and Sox2 [43]. Interestingly, a feedback regulation was also identified, which showed that Esrrb could activate *Klf4* expression by targeting its promoter [43]. However, these two processes were not equally important to pluripotency regulation. Ectopic expression of *Esrrb* could rescue the triple knockdown of *Klf2*, *Klf4* and *Klf5*, preventing induced ESC differentiation; whereas, none of these Klfs could rescue the differentiation caused by *Esrrb* depletion [43]. 

Being a binding partner of Oct4 and Sox2 as well as an upstream regulator of *Nanog*, Klf4 apparently sits in the core regulatory circuitry within the entire transcription network for maintaining pluripotency. Hence it is not surprising that Klf4 in fact cooperates with Oct4 and Sox2 to activate many other pluripotency genes [77], including *Lefty1* and *Sox2* [83]. Consistently, genome-wide binding map analysis revealed that a large proportion of Klf4 binding targets overlapped with Oct4-Sox2-Nanog co-binding loci [29,84], which extended the target gene list of Klf4.

On the other hand, expression of *Klf4* itself was found to rely on LIF/Stat3 signaling that was activated by supplementing Lif in ESC culture medium [85]. *Klf4* is a direct downstream target of LIF/Stat3, and its ectopic expression in ESCs increased their resistance to differentiation upon LIF withdrawal [85]. These findings explained the requirement of LIF for ESC maintenance. Interestingly, using serum-free medium for ESC culture revealed that, although *Klf2*, *Klf4* and *Klf5* play a redundant role in ESC maintenance and iPSC generation, they actually respond differently to the LIF/Stat3 signal [86]. Both *Klf4* and *Klf5* were downstream targets of LIF/Stat3. They responded to LIF stimulation and activated downstream pluripotency genes, such as *Nanog* and *Sox2*, to maintain ESC self-renewal [85,87,88]. *Klf2*, however, was a direct target of Oct4, showing no obvious response to LIF [86]. In serum-free culture conditions, where LIF is dispensable, Klf2 functioned to activate Klfs downstream genes for maintaining ESC self-renewal [86]. Given their important positions in pluripotency maintenance, both Klf2 and Klf4 were able to reinstate naïve pluripotency in EpiSCs derived from post-implantation embryos [86]. 

Partially due to the functional redundancy among Klfs, no defect in ICM formation was observed in *Klf2* and *Klf4* null mouse embryos. Instead, *Klf4*^−/−^ mice exhibited neonatal defects [89], whereas mouse embryos lacking *Klf2* showed complex defects in hemodynamic responses and died approximately at E12.5–14.5 [90,91]. Targeted disruption of *Klf5* revealed a defect in ICM, which led to failure of ESC derivation and early embryonic lethality around implantation stage. *Klf5*^−/−^ ESCs showed increased spontaneous differentiation, whereas overexpression of *Klf5* could maintain ESC in the absence of LIF [88]. Collectively, Klf2, Klf4 and Klf5 possess unique properties besides their structural similarity and functional redundancy. 

## 5. Spalt-Like (Sall) Family

Spalt-like family is a class of zinc finger proteins with four known members: Sall1-Sall4. They are evolutionarily conserved from Drosophila to human. *Sall4* predominantly expressed in ICM of blastocysts [92] and germ line in adult tissues [93]. Elimination of *Sall4* is embryonically lethal due to the failure of ICM formation [94] and *Sall4* heterozygous mutant mice exhibited anorectal anomalies and exencephaly [95,96]. Consistently, in the human population, mutation on *Sall4* gene leads to an autosomal dominant disorder termed Okihiro syndrome, which is associated with forehead malformation [96].

Mouse *Sall4* encodes two isoform proteins Sall4a and Sall4b [97], in which Sall4a has eight zinc finger domains and Sall4b has only three [98]. The expression of both isoforms is specific to ESCs. Although *Sall4a* is expressed with a higher abundance, *Sall4b* is the isoform crucial to the maintenance of pluripotency in ESCs [97].

Studies have showed that Sall4 bound to the highly conserved distal enhancer upstream of mouse *Oct4* promoter and modulated *Oct4* expression to maintain ESC pluripotency [99]. Subsequent research suggested that Sall4 was likely functional through forming a protein complex with other core pluripotency factors. Sall4-Oct4 interaction was first unraveled by a proteomic study in mouse ESCs [100], and it was found to play a critical role in controlling *Oct4* expression. In a recent study, direct protein-protein interactions of both Sall4-Oct4 and Sall4-Sox2 were demonstrated by pull-down assay in mouse ESCs [101]. Either Sall4-Oct4 or Sall4-Sox2 complex was found to assemble on Oct-Sox elements for gene activation in mouse ESCs; and Sall4-Oct4-Sox2 triple target sites were enriched in well-established pluripotency genes [101]. In addition to Sall4-Oct4-Sox2, Wu *et al.* reported Sall4 could physically interact with Nanog through its *N*-terminal region. Nanog and Sall4 co-occupied both *Nanog* and *Sall4* enhancer regions in ESCs and activated the gene expression [102]. The direct interaction of Sall4 and Nanog was later confirmed by the Nanog -centered proteomics analysis [12,65] and supported by genome-wide ChIP-seq assay, showing that Sall4 and Nanogco-occupied many binding sites in ESCs [102]. Besides Oct4, Sox2 and Nanog, proteomics studies suggested that Sall4 could also associate with other transcription factors, such as Esrrb, Dax1, MTA2 (NuRD complex component) and Nac1 in mouse ESCs, and linked to TGF-β and WNT signaling through interacting with Usp9X and Cxxc5 [100]. 

One study has systematically examined the effect of several important ESC transcription factors by global gene profiling analysis upon their depletion [103]. Two profiling paths were altered upon repression of these factors. One path was through the repression of either *Oct4* or *Sox2*, and the other path was altered by the repression of either *Esrrb*, *Sall4*, *Nanog* or *Tcfap4* [103], demonstrating Sall4 indeed belongs to an accessary, yet crucial, factor of pluripotency. In line with the intensive connection identified between Sall4 and the pluripotency network, a study demonstrated that ectopic expression of *Sall4* could promote somatic cell reprograming [104].

Several studies explored the mechanism that mediates Sall4’s function in regulating pluripotency. Evidence has suggested that Sall4 governs ESC self-renewal through transcriptional repression, possibly by interacting with different DNA methyltransferases (Dnmts) [105], binding to cell cycle regulator cyclin D1 [106], or competing with Oct4 and acting as an antagonist to Oct4-mediated activation of gene expression, such as *Sall1*–*Sall3* [100,107]. 

## 6. Fork Head/Winged Helix (Fox) Transcription Factors

Fox family transcription factors possess a characteristic butterfly-like FOX domain responsible for DNA binding [108]. They are grouped into FoxA–FoxS, each of them functioning in distinct developmental processes. Here, we will discuss some of the members that are involved in pluripotency regulation. 

*FoxD3* (previously named *Genesis*) is required for maintenance of early embryonic cells. A loss of *FoxD3* in mouse embryo led to embryonic lethality due to massive programmed cell death in epiblast cells. The ICM of the *FoxD3*^−/−^ embryo failed to expand and it could not give rise to ESCs in culture [109]. *FoxD3* is also required for the formation of trophoblast lineage. *FoxD3*^−/−^ mouse embryos did not express the trophoectoderm marker *Cdx2* and resulted in failure to generate trophoblast stem cells [110]. Similarly to that in mouse, *FoxD3* expression was detected in human blastocysts and ESCs [111,112]. However, discrepancy exists, as literatures also showed the absence of *FoxD3* in human ESCs [113]. 

Genetic deletion of *FoxD3* in mouse ESCs via a conditional knockout strategy was found to cause no change in proliferation rate, but led to increased apoptosis and decreased ability to self-renew [114]. Differentiation analysis showed that FoxD3 was essential in repressing differentiation towards mesoderm and endoderm lineages for maintaining pluripotency [114]. Similarly, FoxD3 was found to be necessary for human ESC maintenance through a similar differentiation repressing mechanism [115]. At molecular level, it has been reported that FoxD3 cooperated with Oct4 to regulate *Nanog* expression in ESC [116]. On the other hand, *FoxD3* was also a downstream target of Oct4 [117] and, like many other ESC transcription factors, FoxD3 could auto-regulate itself. High levels of FoxD3 could produce a locally inhibitory transcriptional effect at its promoter [117].

Other Fox members were also accounted for ESC maintenance. *FoxH1* and *FoxO1* mRNAs were detected in human ESC [118]. Loss of *FoxH1* led to a wide range of developmental defects [119]. To date, *FoxH1* has not been shown to regulate any target gene directly. Its function is possibly involved in modulating dynamic patterns of *Nodal* expression during early mouse development [120,121]. FoxO1 was found to be essential for human ESC maintenance, through occupying and activating *Oct4* and *Sox2* promoter [122]. FoxO1 protein was primarily phosphorylated and negatively regulated by Akt serine/threonine protein kinase in various cellular contexts [123]. However, this is not true in human ESCs [122]. Interestingly, unlike other critical regulators in stem cell control, genetic deletion of *FoxO1* did not cause early developmental defects in mice [124].

In addition to the above Fox members, an alternatively spliced form of *FoxP1* was discovered to be expressed specifically in undifferentiated ESCs [125]. The ES-specific *FoxP1* contains additional exon 18b (equivalent to exon 16b in mouse FoxP1). Inclusion of exon 18b changed the DNA-binding preference of *FoxP1* from promoting to inhibiting differentiation genes, thus favoring the maintenance of ESC and facilitating reprogramming [125]. Both ectopic expression of non-ES form *FoxP1* and silencing of the *FoxP1-ES* inhibited efficient reprogramming [125]. Complete deletion of *FoxP1* in mouse even disrupted the establishment of specific cell types [126] and resulted in early embryonic lethality at E14.5 [127]. 

## 7. Zinc Finger Proteins

### 7.1. Zfp206 (Zscan10)

*Zfp206* (*zinc finger protein 206*) is highly expressed in both mouse and human ESCs and is down-regulated upon differentiation [118,128,129], suggesting that *Zfp206* functions in maintaining pluripotency. ESCs stably overexpressing *Zfp206* appeared morphologically normal and were resistant to retinoic acid-induced differentiation. In support of this observation, silencing of *Zfp206* was not sufficient to induce differentiation in ESCs, but these cells were susceptible to differentiation induction [129].

Localization to the nucleus, presence of zinc finger domain and a SCAN domain in Zfp206 suggested itself as a transcriptional factor. Indeed, Zfp206 could activate the promoters of *Nanog* and *Oct4* as well as its own promoter [129]. In addition, genome-wide ChIP-chip assay also identified *Klf2*, *Klf4*, *Klf5*, *Zfp281* and *Sall4* as the direct targets of Zfp206 [130]. The binding consensus sequence of Zfp206 has been predicted to be a perfect palindrome (GCGCATGCGC), suggesting Zfp206 might bind to DNA as a homodimer [130]. As with many other pluripotency regulators, reciprocal regulations by core transcription factors were also present in *Zfp206*. Oct4 and Nanog binding sites in *Zfp206* promoter/intron were identified, and these sites were required for Oct4/Sox2 mediated-activation of *Zfp206* [9,10,131], indicating that *Zfp206* was a direct target of Oct4 and Nanog. 

Zfp206 also contains a SCAN domain, which is known to be specific to vertebrates and highly conserved to mediate protein-protein interactions [132]. In line with this notion, physical interaction of Zfp206 with other pluripotency factors has also been demonstrated. Zfp206 directly interacted with Oct4 and Sox2 [130]. Zfp206 shared a significant number of targets with Oct4 and Sox2, which included genes that play an essential role in ESC (such as *Oct4*, *Jarid1*, *Klf2*) and in mouse development (such as *Hoxb13*, *Meis1*, and *Pax6*) [130]. Zfp206 has also been found to interact with other SCAN domain containing proteins such as Zscan4 and Zfp110 [133]. Zscan4 is known as a regulator of telomere extension and genomic stability in ESCs [134], while Zfp110 is involved in programmed cell death in the mouse embryonic neural retina [135]. Collectively, these results demonstrated that Zfp206 is an integral component of pluripotency network. 

### 7.2. Rex1 (Zfp42)

*Reduced expression 1* (*Rex1*, or *Zfp42*) encodes a protein containing four Cys–His-type zinc-fingers. *Rex1* is exclusively expressed in early embryos, including both trophectoderm and ICM, and is selectively restricted to germ cells during later development [136]. In both human and murine ESCs, expression of *Rex1* rapidly decreases upon differentiation; thus it has been widely used as a marker for pluripotent stem cells [136,137]. Consistent with *Rex1*’s specific expression, several studies have demonstrated that it was a direct target of the core pluripotency genes in ESCs. There were binding sites for Oct4, Nanog, Dax1, NacI, and Klf4 in the *Rex1* promoter [28,138]. Nanog stimulated *Rex1* expression by directly activating the *Rex1* promoter, while Oct4 and Sox2 activated or repressed the *Rex1* promoter, depending on the cellular environment [139,140]. However, *Rex1*^−/−^ mice showed no defects in early development [141] and *Rex1*^−/−^ ESCs could self-renew robustly and remain pluripotent [142,143]. 

Genome wide binding site mapping showed that, unlike other pluripotency factors, which often form a feed-forward auto-regulatory loop with the core factors, Rex1 target sites were largely grouped with c-Myc binding sites, but not overlapped with those recognized by core pluripotency factors [28]. A later study found that Rex1 directly targeted and inhibited the transcription of *Xist* to maintain the X activation in mouse ESCs [144]. Rex1 was also implicated in establishing the epigenetic modifications required for maintaining allele-specific DNA methylation in imprinting genes, such as *Peg3* and *Gnas* domains [145]. In addition, Rex1 was found to up-regulate *cyclin B1/B2* expression, which subsequently activated cyclin B/CDK1 and induced the phosphorylation of DRP1, leading to mitochondrial fission that met the energy needs of human ESC via anaerobic pathways [146]. 

Collectively, these findings suggest that Rex1 may not be crucial to the maintenance of the core transcription network, but it plays auxiliary roles in maintaining other cellular features in ESCs.

### 7.3. Zscan4

*Zscan4* (*zinc finger and SCAN domain containing 4*) is a novel gene identified in 2007 [147]. The expression of *Zscan4* is restricted to late 2-cell stage embryos and ESCs [134,147,148,149]. Notably, there were only a small fraction (1%–5%) of undifferentiated ESCs expressing *Zscan4* at a given time [147,148], but all ESCs had *Zscan4* expressed at least once within nine cell passages [134]. Knockdown of *Zscan4* had no direct effect on ESC self-renewal and pluripotency, but induced telomere shortening and subsequently culture crisis [134]. By a telomere chromosome orientation FISH (CO-FISH) assay, it was concluded that transient expression of *Zscan4* promoted telomere recombination, leading to telomere elongation [134]. Zscan4 also reduced the DNA damage response and enhanced the efficiency of iPSC generation [150], generating iPSCs with higher genome stability [150]. 

ESCs have the capacity to maintain their high potency for many cell passages [151], however, loss of potency is still observed in long-term culture. In a recent finding, the developmental potency of ESC was found to be rapidly restored by the transient activation of *Zscan4* [152]. Analysis on Zscan4-dependent genes in ESC or during reprogramming suggested that modulation of *Zscan4* level did not alter the transcriptome dramatically [153]. To date, it remains unclear how pluripotency factors control the transient expression of *Zscan4* and what is the molecular mechanism that mediate the function of Zscan4 in ESCs. 

### 7.4. Other Zinc Finger Proteins

Besides the above zinc finger proteins, several other identified zinc finger proteins, including Zfp296, Zfp281 and Zfp143, have also been proposed to interact with the core pluripotency transcription factors. Zfp296 was shown to bind to CR4 in *Oct4* distal enhancer to activate *Oct4* transcription. Addition of Zfp296 also enhanced reprogramming efficiency [154]. Zfp281 could physically interact with Oct4, Sox2, and Nanog [65,155]; and it activated *Nanog* expression directly through binding to a motif in close proximity with the Oct4-Sox2 binding site in the *Nanog* proximal promoter [155]. Zfp143 regulated *Nanog* expression through physical interacting with Oct4 and modulating its binding to *Nanog* promoter [156]. Currently, there is still a lack of thorough investigation to further elaborate the functional role of these Zfps as well as to examine their importance in overall pluripotency establishment and maintenance. 

## 8. Developmental Pluripotency-Associated Genes (Dppa)

Dppa family is a group of genes identified for their exclusive expression in cells within the pluripotent cycle throughout development, including blastomeres in embryos at cleavage stages, ICM of blastocysts, developing germ cells, and ESCs in culture [157,158,159]. Five *Dppa* genes (Dppa1–5) have been shown to be expressed exclusively in these undifferentiated cells, but their functions are poorly characterized. Since the physiological role of *Dppa1* has not been evaluated [158], here we only summarized the current understanding on the functions of *Dppa2*–*5* in pluripotency regulation.

*Dppa2* and *Dppa4* are closely linked genes encoding proteins containing a putative nuclear SAP (SAF-A/B, Acinux and PIAS) motif, which is responsible for DNA/RNA-binding and is involved in chromatin modification [157,160]. Although both *Dppa2* and *Dppa4* were expressed in ICM, developing germ line and ESCs, they exhibited different dynamic expressions during the development [157]. Depletion of either *Dppa2* or *Dppa4* by shRNA resulted in differentiation of ESCs [42,161,162]; whereas, knockdown *Dppa2* also resulted in decreased proliferation of ESCs [161]. These findings suggested that Dppa2 and Dppa4 play essential roles in the maintenance of ESC pluripotency *in vitro*; however, the underlying mechanism and their connection to the core pluripotency regulatory circuitry remain unclear.

*Dppa3* (*Stella*) is a definitive marker of the germ cell lineage, but its expression was also observed in preimplantation embryos and ESCs [163,164]. Similarly, *Dppa3* encodes a protein with a SAP-like domain and a splicing factor motif-like structure, suggesting it functions in chromosomal organization or RNA processing [165]. *Dppa3*^−/−^ mice generated via targeted mutation were apparently normal at birth, but the adult females displayed severely reduced fertility due to a lack of maternal Stella-protein in their oocytes [165]. In ESCs, expression of *Dppa3* has been reported to be heterogeneous in both mouse and human ESCs [164,166]. Single cell gene expression analysis showed that Dppa3 (+) mouse ESCs were similar to the ICM, whereas Dppa3 (−) cells resembled the epiblast cells at a later stage [167]. The Dppa3 (+) and Dppa3 (−) states were exchangeable, but Dppa3’s function in ESCs still remains obscure due to a lack of characterization on *Dppa3^−/−^* ESCs. 

*Dppa5* (or *embryonic stem cell-specific gene 1*, *Esg1*; or *ESC associated transcript 2*, *Ecat2*) encodes a KH-domain containing protein, and it is specifically expressed in early embryos, germ cells, and ESCs. *Dppa5*^−/−^ mice generated via gene targeting developed normally and fertile [168]. *Dppa5*^−/−^ ESCs demonstrated normal morphology, proliferation, and differentiation [168]. Hence, despite its specific expression, Dppa5 is dispensable for the establishment and maintenance of pluripotency. 

## 9. T-box 3 (Tbx3)

Tbx3 is a member of the T-box family. In mice, homozygous mutations were embryonic lethal due to various developmental defects [169]. Studies have shown that Tbx3 was essential in the maintenance of mouse ESC self-renewal. Depletion of *Tbx3* resulted in the loss of pluripotency and differentiation [42,170], while overexpression of *Tbx3* was found to be sufficient to maintain the undifferentiated state in the absence of LIF [87], similar to that in *Nanog*-overexpressing cells [22,23]. 

In mouse ESCs, expression of *Tbx3* was partially activated by PI3-kinase but inhibited by MAP-kinase [87]. Furthermore, *Tbx3* promoter was directly bound by both Nanog and Tcf3, suggesting its regulation by WNT signal pathway or through GSK3β kinase [170]. On the other hand, Tbx3 predominantly stimulated *Nanog* expression, meanwhile targeting ESC factors *Oct4*, *Sox2*, *Sall4*, *Lefty1*, *Lefty2* and *Zfp42*, as well as reprogramming factors *Klf2*, *Klf4*, *Klf5*, *n-Myc* and *c-Myc* [87,170]. Hence, it is not surprising that expression of *Tbx3* during somatic cell reprogramming could improve the overall quality of iPSCs [170]. 

In human ESCs, *Tbx3* overexpression promoted proliferation by repressing the expression of cell cycle regulators *NFκBIB* and *p14^ARF^* [171]. During differentiation, *Tbx3* depletion resulted in decreased neural differentiation [171]. Collectively, these findings indicated that Tbx3 plays an important role in maintaining pluripotency in ESCs. 

## 10. Germline Genes

Germ stem cells derived from mouse embryonic primordial germ cells are pluripotent and highly resembling ESCs [172], suggesting a close relationship between germ cells and ESCs. Indeed, many ESC markers are known to be enriched in developing germ cells, such as the aforementioned *Oct4*, *Dppa4*, and *Nr5a2* [173,174]; meanwhile, germline specific genes, such as *Prdm14*, *L1td1* and *Utf1*, have been implicated in ESC maintenance. Here we will review studies in this area.

### 10.1. Prdm14

Prdm14, a PR domain-containing transcriptional regulator, was identified as one of the major determinants of human ESC identity in a whole-genome RNAi screen [175]. Prdm14 directly regulated the expression of *Oct4* through its proximal enhancer [175]. In addition, deletion of *Prdm14* in human ESCs resulted in increased expression of differentiation genes [175,176], whereas ectopic expression of *Prdm14* suppressed the expression of differentiation genes in embryoid bodies [176], suggesting that Prdm14 also acts as a transcriptional repressor in regulating pluripotency in human ESCs. Indeed, Prdm14 facilitated iPSC generation by repressing differentiation-related genes [175,177], in particular, the mesenchymal genes during the initiation stage [175]. Furthermore, its repressive role was proved to be mediated through cooperation with polycomb repressive complex 2 (PRC2) [177,178]. 

Interestingly, *Prdm14* homologue in mouse is a determinant factor for germline formation and maintenance [179]. Depletion of *Prdm14* did not cause obvious differentiation of mouse ESCs [175,178,180]. Instead, it is involved in ensuring naive pluripotency through repressing the FGF signaling pathway, or *de novo* DNA methyltransferases activities in ESCs [178,180]. 

### 10.2. L1td1 (Ecat11)

*L1td1* (*line-1 type transposase domain-containing protein 1*, *or ES cell associated transcript 11*, *Ecat11*) encodes a RNA-binding protein that is abundantly expressed in mouse ESCs and is suppressed upon differentiation [181,182]. Despite this specific expression, *L1td1*^−/−^ mice grew normally and were fertile. Moreover, *L1td1* was dispensable for both proliferation and pluripotency of ESCs. More importantly, iPSCs could be established from *L1td1*^−/−^ fibroblasts [181].

Surprisingly, L1td1 has a critical function in human ESC maintenance. L1td1 co-localized and interacted with Lin28 via binding to common RNAs, and it was implicated in modulating the level of *Oct4* expression [182]. Depletion of *L1td1* resulted in immediate down-regulation of *Oct4* and *Nanog*, and subsequently induced differentiation. In return, Oct4, Sox2, and Nanog proteins bound to the promoter of *L1td1* and regulated its expression level [182].

### 10.3. Utf1

*Utf1* (*undifferentiated embryonic cell transcription factor 1*) is expressed during early embryonic development in the cells of ICM and epiblasts; its expression is then rapidly restricted to primordial germ cells of developing embryos [183] and gonads in adults [183,184,185,186]. On one hand, Utf1 has been implied to maintain the proliferation rate of ESCs, and was required for proper teratoma formation [187,188]. On the other hand, Utf1 was shown to be dispensable for self-renewal, but ESCs with reduced Utf1 could not differentiate properly [188]. Promoter analysis has found that the mouse *Utf1* is directly regulated by Oct4 and Sox2 [189]. Utf1 has also been implied to function in chromatin-associated transcriptional repression [188]. A recent study demonstrated that Utf1 buffered the poised states of bivalent genes through limiting PRC2 loading and histone 3 lysine-27 (H3K27) trimethylation, thus set activation thresholds for differentiation-promoting genes in ESCs [190]. 

## 11. Conclusions

To summarize, the key transcription factors Oct4, Sox2 and Nanog form the core pluripotency circuitry with their cooperative autoregulation in ESCs. These core transcription factors work in combination to further activate other pluripotency-related genes through feed-forward regulatory loops, or to repress differentiation-promoting genes via feedback inhibitory regulations. Encompassing this core circuitry, ancillary transcription factors either provide feed-forward regulations to strengthen and stabilize the pluripotency network, or branch out from the regulating transcription network to exerting other cellular functions required for maintaining ESC identity (Table 1). 

Accumulating knowledge about the transcriptional network in ESCs has provided a comprehensive elucidation of molecular regulations underlying pluripotency maintenance; however, challenges still remain to fully understand the unique property in ESCs. First, intensive investigation is required to elaborate on the complex regulatory roles of intrinsic/extrinsic signals and their connections to the transcriptional network in ESCs. Since the establishment of ESC, various signaling pathways have been implicated in regulating ESC identity. These include LIF, BMP, FGF and TGFβ/Activin, WNT, SHH, retinoic acid signaling and much more. Although extensive research has been performed to analyze each pathway individually, many conclusions remain controversial and the combinatory effect of these signals remains unclear. In particular, the drastic difference between mouse ESCs and human ESCs has brought about a very puzzling situation, suggesting that the current understanding of signaling molecules is far from sufficient for manipulating ESC differentiation into desired cell types. Second, accumulating evidence has suggested that the pluripotent state is governed by a stringently controlled yet highly dynamic transcriptional network. Transcriptional regulations of individual components under this network are often associated with epigenetic modification and chromatin remodeling, and their activities are subjected to fine-tuning by post-transcriptional or post-translational modulations. Given the massive interactions among various types of regulators, it remains to be a challenging area of research to provide precise understanding of pluripotency regulation, despite the existence of advanced computation modeling technology. Collectively, investigation into the pluripotency regulations with more integrative analyses is required, in order for us to consolidate our understanding as well as to harness the full potential of human ESCs/iPSCs in various research and therapeutic applications. 

**Table 1:** Connections between ancillary and core pluripotency transcription factors.

Gene	Transcriptional interaction with core factors	Reference
*Esrrb*	Interacts with Oct4, Nanog, Sox2 and Ncoa3;	[41,44,45,46,47,48]
	Activate *Nanog*, *Oct4* and other genes;	
	Target of Nanog and Tcf3.	
*Nr5a2*	Interacts with Dax1 and Rarγ;	[50,51,53,54,56,57]
	Activates *Oct4* and *Nanog*;	
	Direct target of Oct4, β-Catenin and Tcf3.	
*Dax1*	Interacts with Nanog, Oct4 and Nr5a2;	[24,37,45,62,63,64,65,66,68]
	Activate *Oct4* expression;	
	Target of Stat3, Oct4, Esrrb, Sox2, Nr5a2, Nanog and β-catenin.	
*GCNF*	Repress *Oct4* and *Nanog* upon differentiation.	[50,51,69,73]
*Klf4*	Interacts with Oct4 and Sox2;	[43,77,80]
	Activate *Nanog*, *Esrrb* and other genes;	
	Downstream target of LIF/Stat3 signaling.	
*Sall4*	Interact with Nanog, Oct4, Sox2, Esrrb, Dax1, MTA2 and Nac1;	[99,100,101,102,105,106]
	Activate *Oct4* expression;	
	Linked to TGF-β and WNT signaling through Usp9X and Cxxc5;	
	Involved in transcriptional repression, cell cycle regulation (via binding to cyclin D1).	
*FoxD3*	Activate *Nanog* through cooperating with Oct4;	[116,117]
	Target of *Oct4*;	
	High level of FoxD3 can inhibit itself.	
*FoxO1*	Essential for human ESC maintenance;	[122]
	Activate *Oct4* and *Sox2* in human ESCs.	
*Zfp206*	Interact with Oct4, Sox2, Zscan4 and Zfp110;	[9,10,129,130,131,133]
	Activate *Nanog* and *Oct4*;	
	Target of *Oct4* and *Nanog*.	
*Zfp296*	Activate *Oct4*;	[154]
	Enhance reprogramming.	
*Zfp281*	Interact with Oct4, Sox2 and Nanog;	[65,155]
	Activate *Nanog*.	
*Rex1*	Target of Oct4, Sox2, Nanog, Klf4, Dax1 and NacI;	[28,138,144,145,146]
	Maintains X-activation, imprinting, cell cycle, and mitochondrial fission in ESCs.	
*Zscan4*	Overexpression or knockdown *Zscan4* did not alter the transcriptome dramatically;	[134,150,152,153]
	Transient expression of *Zscan4* lead to telomere elongation and can restore the developmental potency of ESC.	
*Dppa2* & *Dppa4*	Essential for ESC maintenance;	[9,157,160]
	Putative target of Oct4.	
*Tbx3*	Activate *Nanog*, *Oct4*, *Sox2*, *Sall4*, *Lefty1*, *Lefty2*, *Zfp42*, *Klf2*, *Klf4*, *Klf5*, *n-Myc* and *c-Myc*;	[87,170]
	Target of *Nanog* and *Tcf3*;	
	Partially activated by PI3-kinase but inhibited by MAP-kinase;	
	Repress the expression of cell cycle regulators *NFκBIB* and *p14^ARF^*.	
*PRDM14*	Activate *Oct4* in human ESCs;	[175,177,178]
	Repress differentiation-related genes;	
	Interact with PRC2 complex to repress gene expression.	
*L1td1*	Interacts with Lin28 to modulate levels of *Oct4*;	[182]
	Target of Oct4, Sox2, and Nanog in human ESCs.	
*Utf1*	Direct target of Oct4 and Sox2;	[188,189]
	Involved in chromatin-associated transcriptional repression.	
